# AI-Based Model Estimation for a Precision Positioning Stage Employing Multiple Control Switching

**DOI:** 10.3390/mi16121305

**Published:** 2025-11-21

**Authors:** Fu-Cheng Wang, Bo-Xuan Zhong, Chi-Wei Wen, I-Haur Tsai, Jia-Yush Yen

**Affiliations:** 1 Department of Mechanical Engineering, National Taiwan University, Taipei 106319, Taiwan; a27845361@gmail.com (B.-X.Z.); t2245058658@gmail.com (C.-W.W.); jyen@mail.ntust.edu.tw (J.-Y.Y.); 2 Department of Energy and Refrigerating Air-Conditioning Engineering, National Taipei University of Technology, Taipei 106344, Taiwan; iht@mail.ntut.edu.tw; 3 Department of Mechanical Engineering, National Taiwan University of Science and Technology, Taipei 106335, Taiwan

**Keywords:** model estimation, artificial intelligence, control switching, PZT, stage

## Abstract

In this paper, we propose a real-time model estimation framework using artificial intelligence techniques and apply it to a piezoelectric transducer (PZT) stage equipped with multiple switching controllers. Conventional fixed controllers often fail to satisfy diverse performance requirements: some achieve smooth but slow responses, while others deliver fast yet oscillatory behavior. To address this limitation, we developed a multi-controller switching mechanism that can select optimal control sequences based on predicted system responses, thereby enhancing overall performance. However, the existing mechanism relies on a nominal plant and neglects variations during operation. To address this problem, we employ the eXtreme Gradient Boosting (XGBoost) algorithm to construct a real-time model estimator, which continuously updates the system model during response prediction, thereby improving prediction accuracy. The corresponding controllers are then adjusted according to the updated models and integrated into the switching mechanism to further enhance performance. Finally, we validate the proposed approach through simulations and experiments.

## 1. Introduction

Piezoelectric transducers (PZTs) are frequently applied in precision engineering due to the high resolution and rapid response. However, inherent nonlinearities can significantly impair positioning accuracy. Therefore, extensive research has focused on modeling and compensation strategies for PZTs. Among these, the Bouc–Wen model has become a prominent framework for characterizing the nonlinear behavior of PZT actuators. For example, Saleem et al. [1] applied particle swarm optimization (PSO) to tune model parameters and achieved a 90% improvement in positioning precision. Gan and Zhang [2] utilized nonlinear least-squares estimation to identify the generalized Bouc–Wen model’s parameters. Sumitha and Anandaraju [3] combined a linearized Bouc–Wen model with Kalman filtering to enhance nanopositioning performance. Ming et al. [4] integrated a modified Bouc–Wen model with feedforward compensation to improve PZT stage control. McCartney et al. [5] proposed a compact PZT model, which can capture the electromechanical coupling and nonlinear effects, thereby improving simulation efficiency and control design.

Control strategies based on PZT models have been extensively explored. For instance, Nafea et al. [6] proposed a hybrid approach that integrates a PSO-tuned proportional–integral–derivative (PID) controller with hysteresis observation for piezo-actuated micro-positioning stages. Shi et al. [7] combined adaptive feedback control with feedforward compensation for enhanced precision in PZT stages. Ahmad [8] introduced a robust digital controller incorporating Bouc–Wen compensation. Al-Jodah et al. [9] employed pole placement with sensitivity shaping, achieving a 91% reduction in positioning error compared to feedforward compensation using an inverse Bouc–Wen model. Makarem et al. [10] presented a data-driven method for automatic PID tuning to address nonlinearities in ultrasonic motors caused by friction and resonance.

Meeting multiple performance requirements simultaneously is challenging for systems with fixed controllers, as they typically cannot satisfy all specifications at once. For example, some controllers deliver fast responses but at the expense of large overshoots, while others provide smooth responses with longer settling times. To address this issue, various real-time controller adaptation techniques have been proposed. Wang et al. [11] introduced data-driven adaptive control to compensate for unmodeled dynamics and achieve high-precision motion control in piezoelectric linear motors. Switching control mechanisms have also been developed to dynamically adjust controller parameters based on operating conditions. For instance, Wolmuth et al. [12] proposed a switched control approach using feedback gain matrices for uncertain systems. Sharma et al. [13] designed an adaptive sliding mode controller with adjustable gains to mitigate over- and under-estimation issues. Wang et al. [14] further developed multiple control switching strategies that select optimal control sequences through response prediction for PZT stages. However, these strategies rely on response predictions based on a nominal plant, which fails to capture model variations during operation. To overcome this limitation, this study applies artificial intelligence (AI) to develop a real-time model estimator, which enables continuous model updates to improve prediction accuracy and overall system performance.

AI techniques have shown remarkable potential across diverse domains. For example, Sarker [15] reviewed the AI-based modeling in various fields. Hosseinzadeh et al. [16] applied AI techniques to develop a predictive maintenance dataset for defect detection in manufacturing systems. Wang and Wang [17] employed eXtreme Gradient Boosting (XGBoost) models to optimize the management of hybrid energy systems. Kong et al. [18] integrated material composition prediction with correlation analysis to forecast material characteristics. Ye et al. [19] explored AI-driven applications for structural health monitoring, maintenance, and management in civil engineering.

In precision positioning, AI techniques have been increasingly employed to enhance model accuracy and control efficiency. For instance, Uralde et al. [20] utilized artificial neural networks to simplify the design of model-based predictive control for PZT actuators. Baziyad et al. [21] utilized support vector machines to compensate for the hysteresis of nanopositioning systems. Artetxe et al. [22] integrated sliding mode control with neural networks to enhance positioning accuracy and robustness for PZT actuators. Dong et al. [23] provided a comprehensive review of neural network-based modeling for time-domain system identification. Building on these advancements, this paper employs the XGBoost algorithm to develop a real-time model estimator, which can improve the PZT stage’s performance by updating the stage models when switching controllers.

This paper is organized as follows: Section 2 introduces the PZT stage and derives the models for robust control design. A multiple control switching mechanism is then developed to improve positioning accuracy by selecting optimal control sequences based on response prediction. To account for model variations during operation, Section 3 proposes an AI-based model estimation method that enhances prediction accuracy by updating the stage models when forecasting system responses. A phase compensator is also designed to eliminate phase lag in feedback control. Section 4 demonstrates the effectiveness of the proposed approaches through simulations and experimental results. Finally, Section 5 concludes the study.

## 2. The Precision Positioning Stage

**Figure 1:**
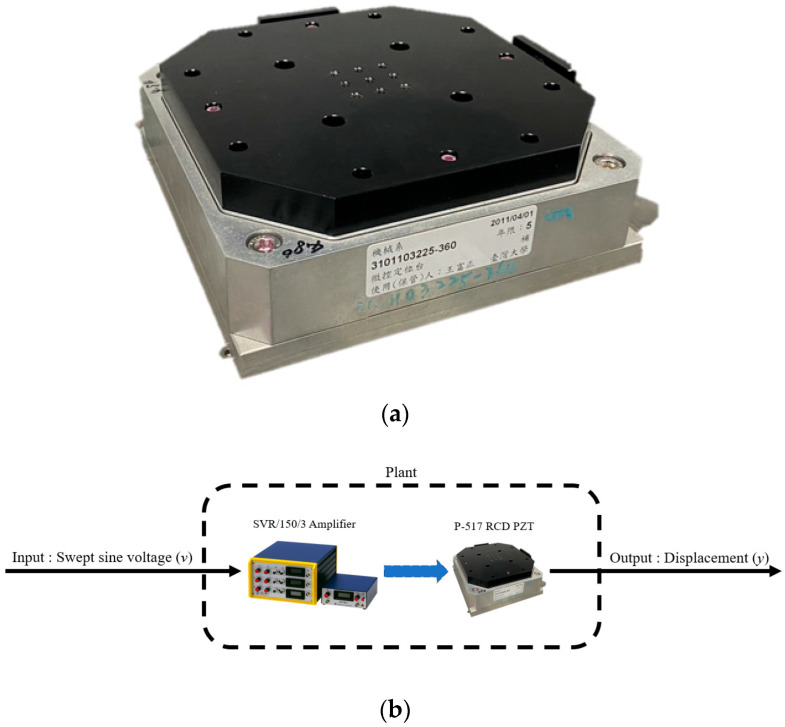
The PZT stage: (**a**) PZT stage; (**b**) Identification.

The PZT stage, shown in Figure 1a, offers a travel of 100 μm with a resolution of 1.22 nm [24]. The detailed specifications are provided in Appendix A.

### 2.1. Stage Identification

The stage models were obtained experimentally, as illustrated in Figure 1b. A swept sinusoidal input *v* was applied, and the corresponding output *y* was recorded to derive stage models. To account for system variations during operation, the identification experiments were repeated ten times, resulting in the following models:

(1) $$\begin{array}{l} G_{1}=\frac{148.0s+3.2\times 10^{5}}{s^{2}+519.3s+4.4\times 10^{4}},\;G_{2}=\frac{149.4s+3.3\times 10^{5}}{s^{2}+524.5s+4.5\times 10^{4}},\;G_{3}=\frac{148.5s+3.2\times 10^{5}}{s^{2}+521.2s+4.5\times 10^{4}},\;\\ G_{4}=\frac{148.9s+3.2\times 10^{5}}{s^{2}+519.3s+4.5\times 10^{4}},\;G_{5}=\frac{148.6s+3.2\times 10^{5}}{s^{2}+520.7s+4.5\times 10^{4}},\;G_{6}=\frac{148.6s+3.2\times 10^{5}}{s^{2}+519.7s+4.5\times 10^{4}},\\ G_{7}=\frac{149.9s+3.3\times 10^{5}}{s^{2}+526.8s+4.5\times 10^{4}},\;G_{8}=\frac{148.5s+3.2\times 10^{5}}{s^{2}+520.8s+4.5\times 10^{4}},\;G_{9}=\frac{148.4s+3.2\times 10^{5}}{s^{2}+521.4s+4.5\times 10^{4}},\;\\ G_{10}=\frac{148.7s+3.2\times 10^{5}}{s^{2}+520.7s+4.5\times 10^{4}}. \end{array}$$

A nominal plant was selected from (1) for control design based on gap metric analysis. Suppose that the nominal plant $G_{0}$ and a perturbed plant $G_{\Delta }$ have normalized coprime factorizations given by:

$$G_{0}(s)=\tilde{M}{\left(s\right)}^{-1}\tilde{N}(s)\mathrm{and}\;G_{\Delta }={\left(\tilde{M}+\Delta_{\tilde{M}}\right)}^{-1}\left(\tilde{N}+\Delta_{\tilde{N}}\right)$$

where $\tilde{M},\tilde{N}\in RH_{\infty }\;$, $\tilde{M}{\tilde{M}}^{*}+\;\tilde{N}{\tilde{N}}^{*}=\;I$, and $\Delta =\left[\begin{array}{cc} \Delta_{\tilde{M}} & \Delta_{\tilde{N}} \end{array}\right]$ [25]. The gap between $G_{0}$ and $G_{\Delta }$ is labeled as $\delta (G_{0},G_{\Delta })$, which represents the smallest perturbation of ${\left\|\Delta \right\|}_{\infty }$ to transfer $G_{0}$ into $G_{\Delta }$. Accordingly, we selected the following nominal plant $G_{0}$ to minimize the maximum gap among all models:

(2) $$G_{0}=\arg\;\underset{G_{0}}{\min}\;\underset{G_{i}}{\max}\delta (G_{0},G_{i}),\forall G_{i}=G_{4}=\frac{148.9s+3.2\times 10^{5}}{s^{2}+521.3s+4.5\times 10^{4}},$$

which corresponds to a gap of $\delta (G_{0},G_{i})\leq 0.0029\;,\forall G_{i}$.

### 2.2. Control Design

**Figure 2:**
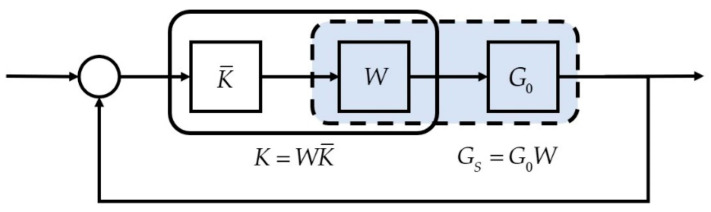
Loop-shaping control design.

We performed loop-shaping control design [26,27], as illustrated in Figure 2. A weighting function *W* was applied to shape the system into $G_{S}=G_{0}W$ for controller design, resulting in a controller $\bar{K}$. Finally, the shaped controller $K=W\bar{K}$ was implemented to control the original plant $G_{0}$.

To satisfy multiple performance requirements, we applied three weighting functions:

(3) $$W^{f}=\frac{30(s+40\pi )}{s(s+15\pi )},\;W^{m}=\frac{15(s+30\pi )}{s(s+40\pi )}\;,\;W^{s}=\frac{5(s+30\pi )}{s(s+50\pi )},$$

to design three robust controllers, as follows:

(4) $$\begin{array}{l} K^{f}=\frac{5.2\times 10^{6}s^{4}+3.8\times 10^{9}s^{3}+8.8\times 10^{11}s^{2}+8.3\times 10^{13}s+2.8\times 10^{15}}{s^{6}+7.4\times 10^{4}s^{5}+6.8\times 10^{7}s^{4}+2.4\times 10^{10}s^{3}+2.7\times 10^{12}s^{2}+8.2\times 10^{13}s},\\ K^{m}=\frac{6.1\times 10^{5}s^{4}+4.6\times 10^{8}s^{3}+1.1\times 10^{11}s^{2}+1.0\times 10^{13}s+3.4\times 10^{14}}{s^{6}+2.9\times 10^{4}s^{5}+2.6\times 10^{7}s^{4}+8.2\times 10^{9}s^{3}+1.0\times 10^{12}s^{2}+4.3\times 10^{13}s},\\ K^{s}=\frac{5.5\times 10^{4}s^{4}+4.2\times 10^{7}s^{3}+1.0\times 10^{10}s^{2}+1.0\times 10^{12}s+3.6\times 10^{13}}{s^{6}+1.1\times 10^{4}s^{5}+8.7\times 10^{6}s^{4}+2.6\times 10^{9}s^{3}+3.2\times 10^{11}s^{2}+1.4\times 10^{13}s}. \end{array}$$

**Figure 3:**
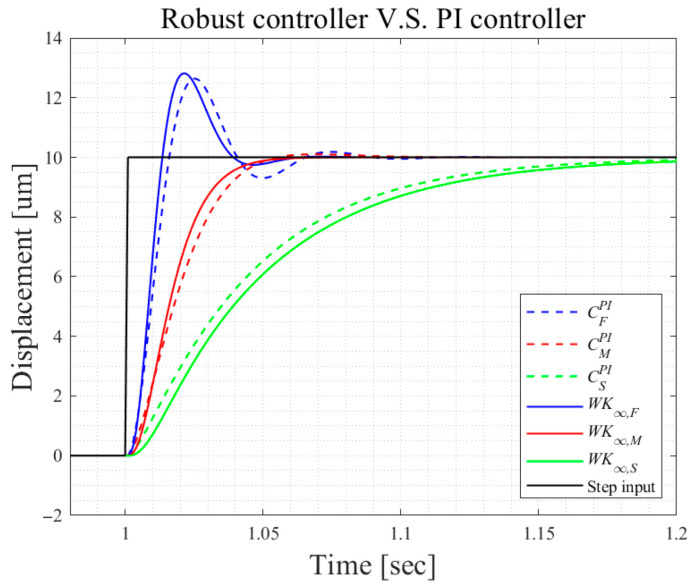
Step responses employing $K^{f},\;K^{m},\;K^{s},\;C^{f},\;C^{m},\;\mathrm{and}\;C^{s}$.

The controllers $K^{f},\;K^{m},\;\mathrm{and}\;K^{s}$ correspond to fast, medium, and smooth responses and exhibit distinct dynamic characteristics, as illustrated in Figure 3. The fast controller $K^{f}$ delivers rapid responses but introduces noticeable oscillations, whereas the smooth controller $K^{s}$ provides slower yet highly stable behavior. The medium controller $K^{m}$ offers a balanced compromise between $K^{f}\;\mathrm{and}\;K^{s}$.

The system robustness is evaluated by the stability margins, defined as:

(5) $$b(G_{0},K)\equiv {\left\|\left[\begin{array}{l} K\\ I \end{array}\right]{\left(I-G_{0}K\right)}^{-1}[I\;G_{0}]\right\|}_{\infty }^{-1}.$$

The system’s internal stability is guaranteed for all $\Delta =\left[\begin{array}{cc} \Delta_{\tilde{M}} & \Delta_{\tilde{N}} \end{array}\right]$ with ${\left\|\Delta \right\|}_{\infty }\leq \varepsilon$ if and only if $b(G_{0},\;K)>\varepsilon$ [28]. The corresponding stability margins of the controllers are $b(G_{0},\;K^{f})=0.3884$, $b(G_{0},\;K^{m})=0.5722$, and $b(G_{0},\;K^{s})=0.6608$. These margins exceed the maximum system gap, ensuring that stability is maintained throughout the experimental conditions.

Because the controllers $K^{f},\;K^{m},\;\mathrm{and}\;K^{s}$ are of sixth order, we further employed PSO algorithms [29] to simplify them into the following proportional-integral (PI) controllers:

(6) $$C^{f}(s)=0.0715+\frac{24.2909}{s},\;C^{m}(s)=0.0407+\frac{7.2924}{s},\;C^{s}(s)=0.0232+\frac{2.9655}{s}.$$

Figure 3 compares the step responses of the original high-order controllers ($K^{f},\;K^{m},\;\mathrm{and}\;K^{s}$) with those of the robust PI controllers ($C^{f},\;C^{m},\;\mathrm{and}\;C^{s}$). First, the results illustrate the trade-offs among different controllers, highlighting the oscillatory nature of the fast controller and the slower yet stable responses of the smooth controller. Second, the PI controllers achieved responses similar to their high-order counterparts. Therefore, the PI controllers will be adopted for subsequent simulations and experiments.

### 2.3. Multiple Control Switching

**Figure 4:**
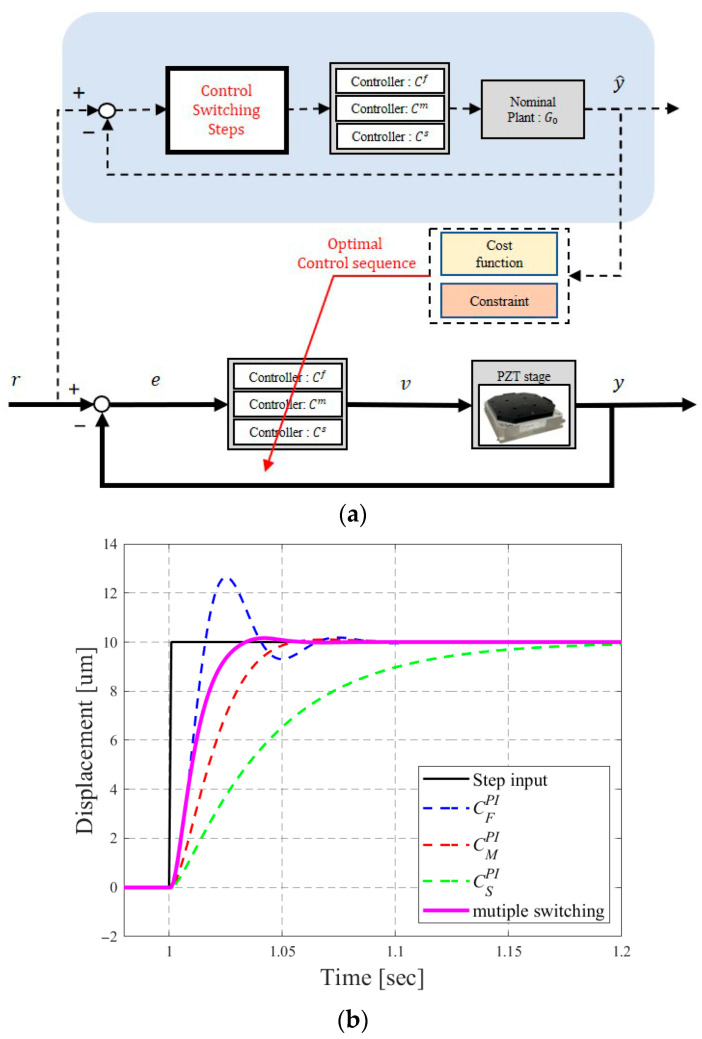
Multiple control switching: (**a**) The mechanism; (**b**) System responses.

Precision systems often demand both rapid and smooth responses, which fixed controllers cannot achieve simultaneously. To address this challenge, Wang et al. [14] proposed a multiple switching control mechanism, as shown in Figure 4a, that determines the optimal control sequence based on predicted system responses.

System responses are predicted based on two parameters: the prediction horizon $H_{P}$ and the switching period $S_{P}$. At time *kT*, future responses are forecasted up to $(k+H_{P})T$, and the root mean square error (RMSE) of all potential responses using $3^{S_{P}}$ control sequences is computed as follows:

(7) $$J={\left(\frac{1}{H_{P}T}\cdot \int_{kT}^{(k+H_{P})T}{\left(r(t)-y(t)\right)}^{2}dt\right)}^{1/2}.$$

Finally, the best control sequence that minimizes *J* is chosen for implementation.

In previous studies [14], we tuned the parameters by iteration. The results indicated that using three controllers with a prediction horizon of $H_{P}$ = 20 and a switching period of $S_{P}$ = 3 achieves comparable performance to configurations with more controllers, larger horizons, and longer switching periods. Therefore, this study adopts three controllers with $H_{P}$ = 20 and $S_{P}$ = 3 in this study. Figure 4b illustrates the system responses under multiple control switching, which combines the advantages of different controllers. Compared with a fixed controller, the switching mechanism effectively mitigates overshoot, shortens settling time, and reduces tracking errors.

## 3. AI-Based Model Estimation

Multiple control switching improves system performance by selecting optimal control sequences through response prediction. However, the current mechanism (Figure 4a) relies on a nominal plant model to forecast responses, neglecting potential variations caused by temperature changes, nonlinearities, disturbances, and noise during operation. To address this limitation, we evaluated several AI algorithms, such as Support Vector Regression and Light Gradient Boosting Machine, and ultimately selected the XGBoost algorithm for its efficient gradient-based computation for real-time applications. The proposed estimator dynamically updates the stage model to enable more accurate response prediction and enhancing overall control performance.

### 3.1. The eXtreme Gradient Boosting Algorithm

**Figure 5:**
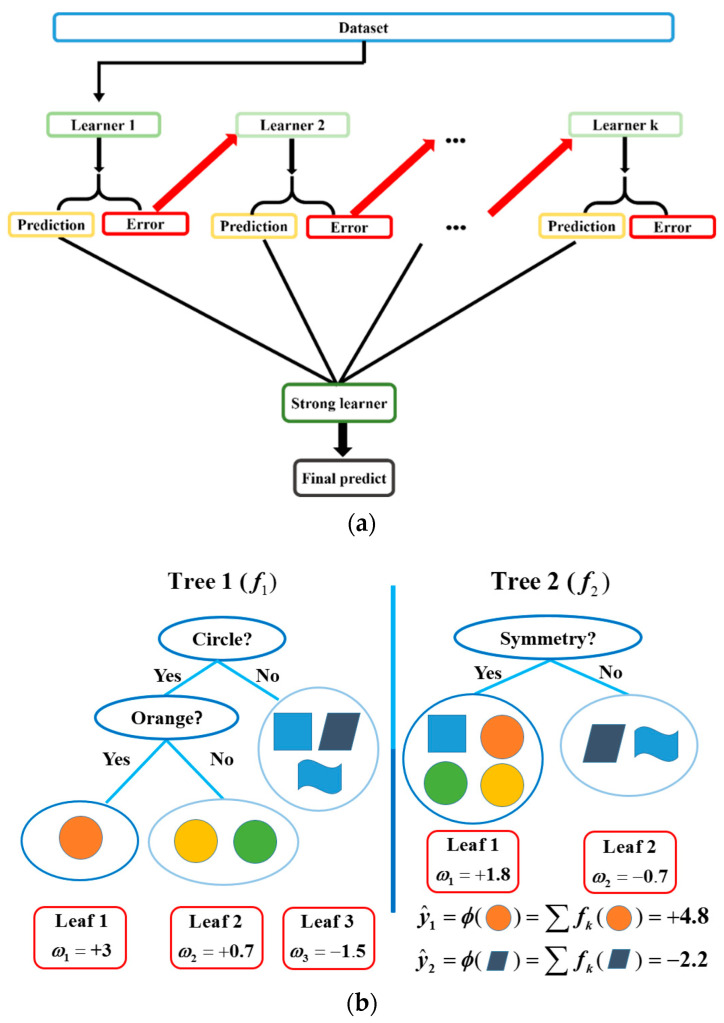
The XGBoost algorithm: (**a**) The boosting algorithm; (**b**) The tree integration.

XGBoost employs a boosting strategy [30], as shown in Figure 5a, which combines multiple weak learners to form a strong predictive model. Each weak learner is represented as a decision tree, sequentially connected to previous trees to correct errors and improve prediction accuracy by adjusting weights. Furthermore, these trees incorporate multiple features to construct an ensemble model capable of delivering highly accurate predictions. For example, Figure 5b demonstrates how each tree classifies features at its nodes to capture distinct characteristics, and the ensemble of trees forms a collective predictive model.

We can derive the XGBoost model using the following indicators:

(8) $${\hat{y}}_{i}=\phi (x_{i})=\sum_{k=1}^{K}f_{k}(x_{i}),f_{k}\in F,$$

where $x_{i}$ denotes feature *i*, ${\hat{y}}_{i}$ is the predicted result of target *i*, $f_{k}(x_{i})$ represents the score of feature *i* on the *k*-th tree, and F={f(x)} is the set of all trees. The objective function can be defined as follows:

(9) $$L(\phi )=\sum_{i}l(y_{i},{\hat{y}}_{i})+\sum_{k}\Omega (f_{k}),$$

in which $l(y_{i},{\hat{y}}_{i})$ is a loss function describing the differences between ${\hat{y}}_{i}$ and the actual result $y_{i}$. $\Omega (f_{k})$ denotes the regularization term, defined as:

(10) $$\Omega (f_{k})=\gamma T+\frac{1}{2}\lambda \sum_{j=1}^{T}\omega_{j}^{2},$$

which controls model complexity through learning weights. Here, γ is a shrinkage factor that penalizes leaf nodes, *T* is the number of leaves, λ limits leaf scores to prevent overfitting, and $\omega_{j}$ represents the score of leaf *j*.

### 3.2. Model Estimation

We applied the XGBoost algorithm to develop a model estimator using the dataset obtained in Section 2.1, which includes the input voltage *v*, the output displacements *y*, and the derived models $G_{i}(s)$ in (1). The model construction involved three steps: data splitting, model training, and model validation.

**Figure 6:**
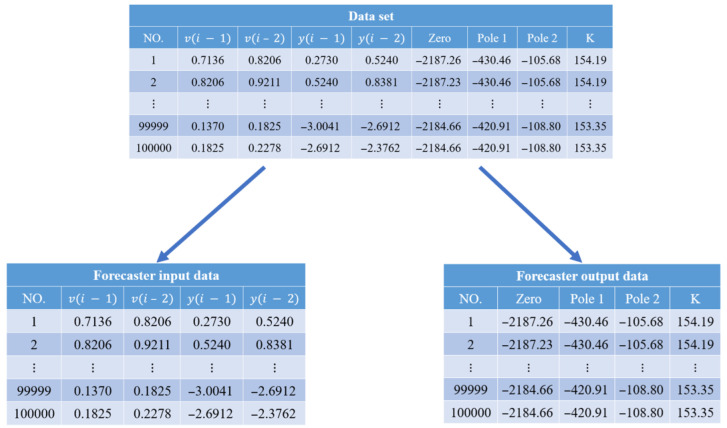
Data splitting.

**Figure 7:**
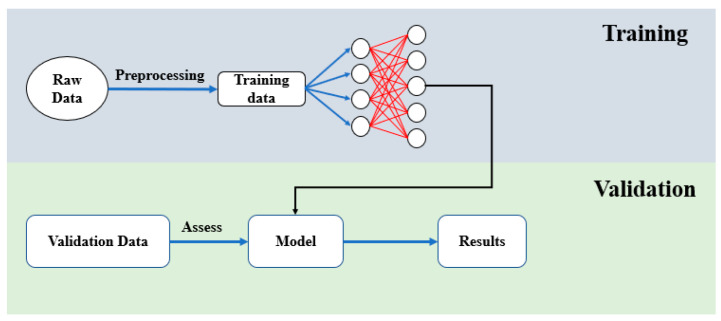
Model training and validation.

(1) Data splitting: For each model $G_{i}(s)$, we labeled its poles, zero, and gains, as shown in Figure 6, along with the corresponding input voltages *v* and output displacements *y*. Since the models in Section 2.1 are second-order strictly proper transfer functions, the input features were set as y(t), y(t−1), y(t−2), v(t), and v(t−1), while the poles, zero, and gain of $G_{i}(s)$ served as the outputs for the model estimator.
(2) Estimator training: The dataset contained 100,000 data points, as shown in Figure 6. Two-thirds of the data used to train the model estimator with the Python (version 3.12.3) command *XGBRegressor* from the XGBoost library, as illustrated in Figure 7. During training, we performed grid search to optimize hyperparameters. The final configuration set the learning rate to 0.4, limited the number of trees to 100, and restricted each tree to a maximum of 16 leaves.
(3) Estimator validation: The remaining one-third of the dataset was used to validate the model estimator, as shown in Figure 6 and Figure 7. The input *v* and output *y* were fed into the trained XGBoost model to obtain an estimated stage model $G_{est}$. The gap between $G_{est}$ and the actual model $G_{i}$ is denoted as $\delta (G_{est},G_{i})$, while the gap between $G_{0}$ and $G_{i}$ is $\delta (G_{0},\;G_{i})$. The estimation is considered effective if $\delta (G_{est},G_{i})$ is significantly smaller than $\delta (G_{0},\;G_{i})$. Validation results show that the average gap for $\delta (G_{est},G_{i})$ is 0.0004, compared to $\delta (G_{0},\;G_{i})=0.0017$, which is substantially larger. This demonstrates that the estimated model $G_{est}$ captures system dynamics more accurately than the nominal model $G_{0}$, confirming the effectiveness of the XGBoost estimator. Furthermore, the estimated gaps are well below the stability margins, ensuring closed-loop stability.

### 3.3. Real-Time Controller Modification

When updating the stage model in the response predictor, the corresponding controllers must also be adjusted as follows:

(11) $$G_{est}C_{est}^{f}=G_{0}C^{f},\;G_{est}C_{est}^{m}=G_{0}C^{m},\;G_{est}C_{est}^{s}=G_{0}C^{s},$$

to maintain consistency in loop transfer functions and system responses. Here, $C_{est}^{f},\;C_{est}^{m},\;\mathrm{and}\;C_{est}^{s}$ represent the corresponding fast, medium, and smooth controllers adapted for the estimated model $G_{est}$.

Since the derived controllers $C_{est}^{f},\;C_{est}^{m},\;\mathrm{and}\;C_{est}^{s}$ are fourth-order, we further simplify them to PI controllers using Hankel singular value analysis [31]. Because the first singular value is significantly larger than the others, the controllers can be reduced to first-order and then simplified as PI controllers, given that the dominant pole is near the origin. Finally, the controllers are converted to discrete-time using the zero-order hold method for implementation.

### 3.4. Phase Compensator Design

**Figure 8:**
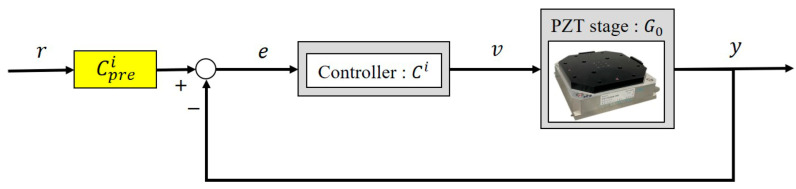
Phase compensator design.

The robust controller design described earlier was based on frequency response shaping, which neglects transient dynamics and phase information. As a result, significant phase lag occurs when inputs vary at high frequencies. To address this issue, we designed a pre-compensator $C_{pre}$ to compensate for phase lag, as shown in Figure 8, where

(12) $$C_{pre}=\frac{k(s+b)}{s+a}.$$

We set *a* = 10,000 and tuned the parameters *b* and *k* based on the closed-loop transfer functions $T=G_{0}C^{i}/(1+G_{0}C^{i})$. The pre-compensator $C_{pre}$ adjusts the closed-loop response to match the desired frequency characteristics $C_{pre}T(j\omega )=1$. For example, for

(13) $$G_{0}(s)=\frac{148.9s+3.2\times 10^{5}}{s^{2}+521.3s+4.5\times 10^{4}}\;\mathrm{and}\;C^{f}(s)=0.0715+\frac{24.2909}{s},$$

the pre-compensator is designed as:

(14) $$C_{pre}(s)=\frac{0.006s+0.881}{0.0001s+1},$$

when s=jω=16πj (8 Hz). Pre-compensators for s=4πj, 10πj, 16πj (2 Hz, 5 Hz, 8 Hz) were derived similarly, as summarized in Table 1. Since the amplifier bandwidth is below 30 Hz, the input frequency was limited to under 10 Hz.

## 4. Simulation and Experiments

**Figure 9:**
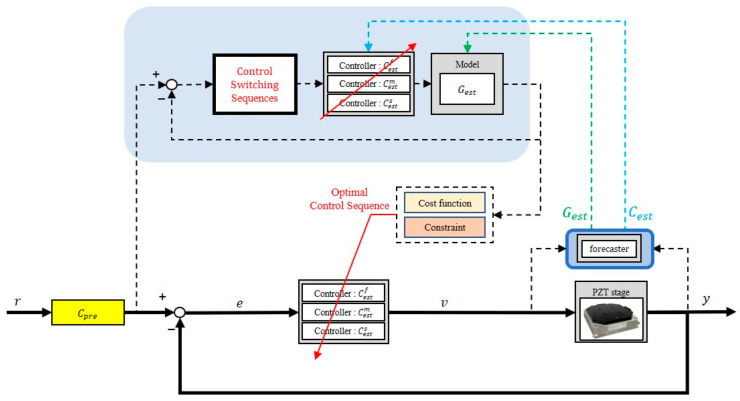
System architecture of the stage control.

We implemented real-time model estimation, control switching, and pre-compensation in the PZT stage, as shown in Figure 9. The multiple-control switching mechanism selected the best control sequence based on response prediction, while the model estimator continuously updated the model during prediction to improve accuracy. Corresponding controllers were then adjusted according to the estimated models.

**Figure 10:**
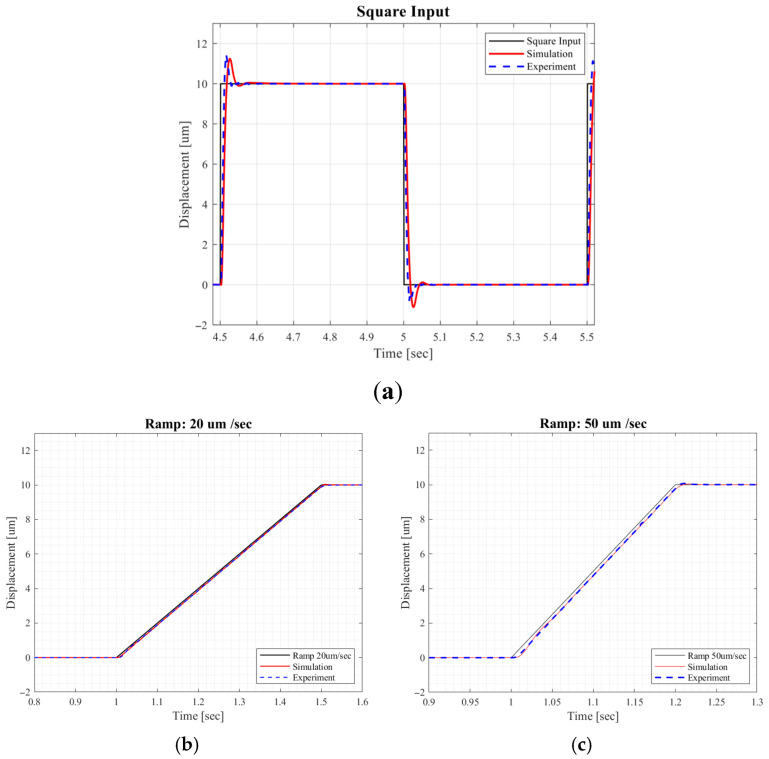

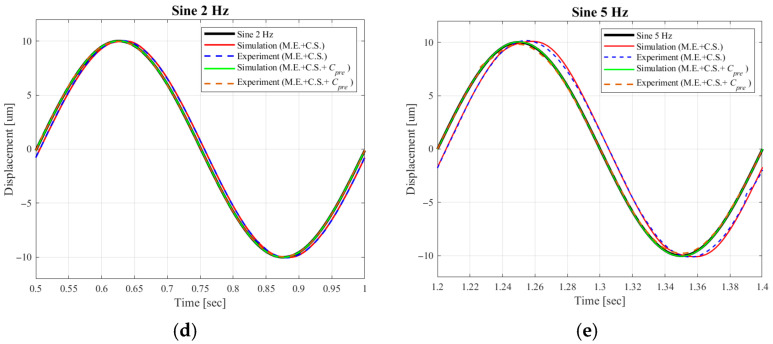
PZT stage responses: (**a**) Square input responses; (**b**) 20 μm/s ramp responses; (**c**) 50 μm/s ramp responses; (**d**) 2 Hz sinusoidal responses; (**e**) 5 Hz sinusoidal responses. (M.E.: model estimation; C.S.: control switching; $C_{pre}$: pre-compensator.).

Simulation and experiments were conducted using the following inputs: (1) a square input; (2) ramp inputs of 20 μm/s and 50 μm/s; and (3) sinusoidal inputs at 2 Hz and 5 Hz. For sinusoidal inputs, a phase compensator was applied to eliminate phase lag. The results are presented in Figure 10 and Table 2.

In the simulation, a plant was randomly selected every 0.5 s, and an estimated model $G_{est}$ was derived using the model estimator. For the square input, rise time, settling time, and RMSE were all improved, although overshoot slightly increased because the switching control optimization targeted RMSE minimization (see (7)). For the ramp inputs, RMSEs were consistently reduced. For sinusoidal inputs, the proposed mechanisms improved phase lag, maximum absolute error (MAE), and RMSE, with the phase compensator providing significant additional enhancement. These results confirm the effectiveness of the proposed approach.

**Figure 11:**
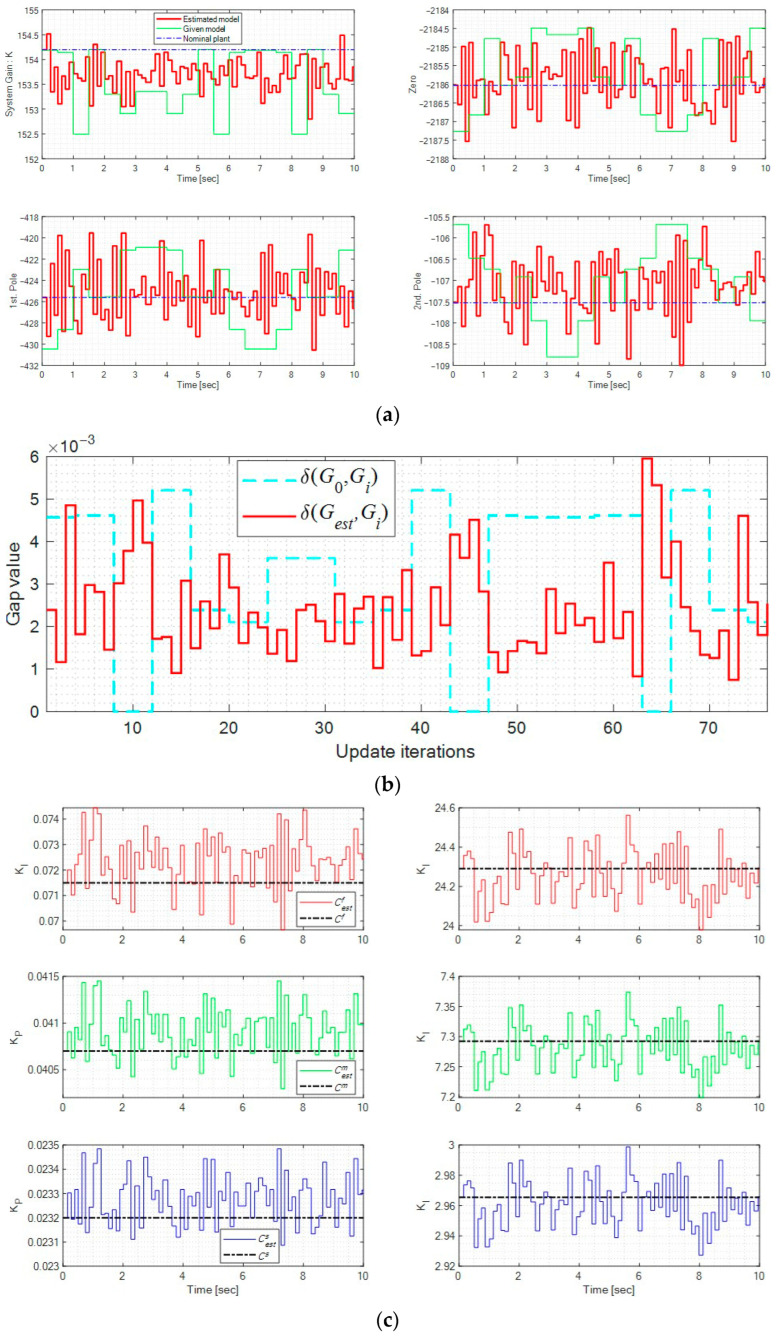
Model estimation (simulation): (**a**) Poles, zero, and gain of the systems; (**b**) System gap variation; (**c**) Real-time controller modifications.

The model estimation process when r=10sin(4πt) is illustrated in Figure 11. First, Figure 11a compares the poles, zeros, and gains of the actual plant $G_{i}$ and the estimated model $G_{est}$, where the model estimator continuously updated the model based on system inputs and outputs. Figure 11b shows the gaps between the two models, where the average gap of the estimated model $\delta (G_{est},G_{i})$ = 0.0004 is significantly smaller than that of the initial model $\delta (G_{0},G_{i})$ = 0.0017—indicating that the estimator generated a more accurate model in real time. Finally, the corresponding controllers ($C_{est}^{f},\;C_{est}^{m},\;\mathrm{and}\;C_{est}^{s}$) were updated in real time according to (6), as shown in Figure 11c.

**Figure 12:**
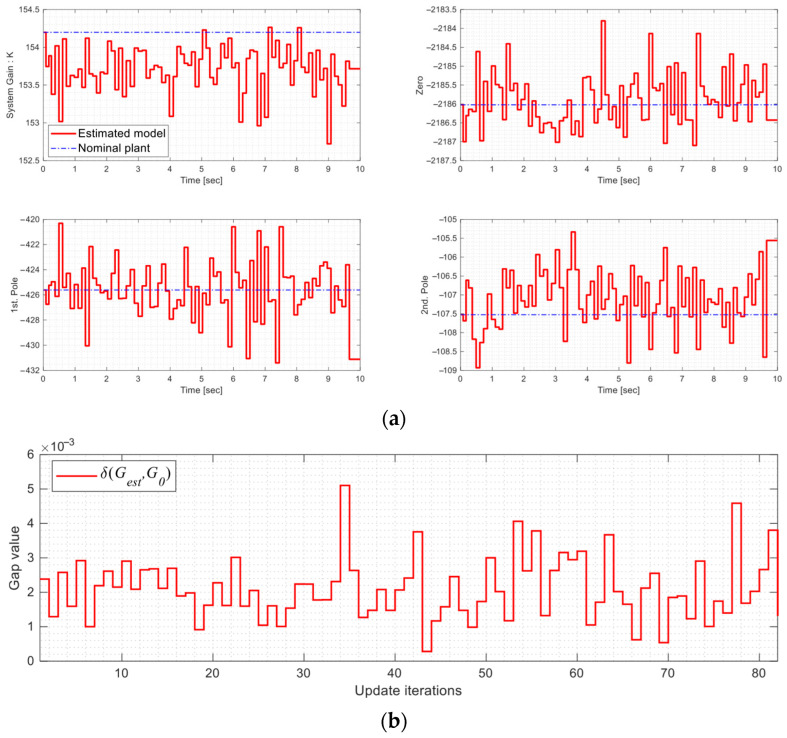

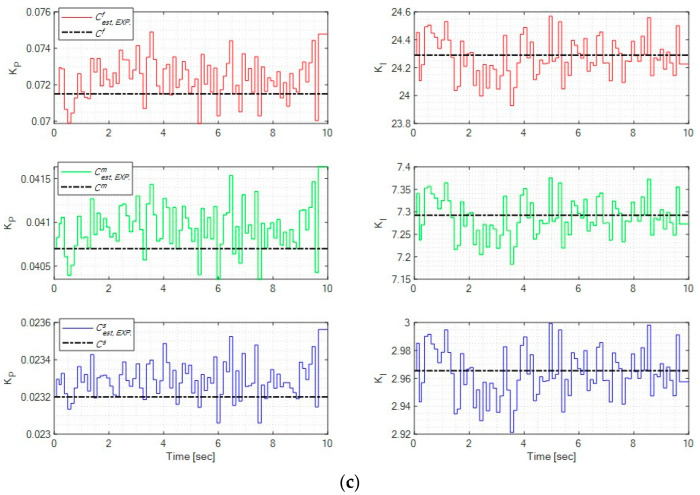
Model estimation (experiment): (**a**) Poles, zero, and gain of the systems; (**b**) Gap of $\delta (G_{est},G_{0})$; (**c**) Real-time controller modifications.

In the experiments, the model estimator continuously updated the estimated model, as illustrated in Figure 12a, to improve response prediction accuracy and overall system performance. According to Table 2, overshoot and RMSE for the square input were reduced, although rise time slightly increased because the optimal switching control prioritized RMSE minimization (see (7)). Similarly, MAE and RMSE for the ramp inputs were improved. For sinusoidal inputs, phase lag, MAE, and RMSE were all reduced, with phase compensators providing significant additional improvement. These results demonstrate the benefits of the proposed mechanisms in practical applications.

However, the improvements observed in experiments were less pronounced than those in simulations, likely due to disturbances and noise in physical systems. To further enhance precision, disturbance observers and noise reduction techniques can be applied in the future.

## 5. Conclusions

This paper presented an AI-based model estimation mechanism and applied it to a PZT stage employing switching control. The switching control mechanism predicted system responses to select optimal control sequences, while prediction accuracy was improved through an XGBoost-based model estimator that updated the model using system input–output data. The estimated model was then applied to update corresponding controllers in real time and design a pre-compensator to mitigate phase lag, particularly under non-steady inputs. Based on model estimation and control adaptation, the proposed approach significantly improved system performance. Finally, we conducted simulations and experiments to validate the merits of the proposed mechanisms in improving the stage performance.

This study demonstrates the potential of AI algorithms in precision positioning systems. The future works include considering the effects of large system variations, such as loading effects, for extended applications. Additionally, AI techniques may be further explored for direct implementation in control system design, as suggested in [32,33]. Integrating reinforcement learning to optimize switching strategies under dynamic environments also represents a promising research direction.

## Figures and Tables

**Table 1:** Pre-compensator designs.

s=4πj	$C_{pre}^{f}(s)=\frac{0.006s+0.993}{0.0001s+1}$	$C_{pre}^{m}(s)=\frac{0.019s+0.983}{0.0001s+1}$	$C_{pre}^{s}(s)=\frac{0.047s+0.975}{0.0001s+1}$
s=10πj	$C_{pre}^{f}(s)=\frac{0.006s+0.953}{0.0001s+1}$	$C_{pre}^{m}(s)=\frac{0.019s+0.895}{0.0001s+1}$	$C_{pre}^{s}(s)=\frac{0.047s+0.843}{0.0001s+1}$
s=16πj	$C_{pre}^{f}(s)=\frac{0.006s+0.881}{0.0001s+1}$	$C_{pre}^{m}(s)=\frac{0.020s+0.735}{0.0001s+1}$	$C_{pre}^{s}(s)=\frac{0.047s+0.609}{0.0001s+1}$

**Table 2:** Tracking performance of the PZT stage.

		Simulation			Experiment		
		C.S.	M.E. + C.S.	M.E. $+\;C.S\;+\;C_{pre}$	C.S.	M.E. + C.S.	M.E. $+\;C.S\;+\;C_{pre}$
Square input	Rising time (s)	0.0117	0.0102	-	0.0065	0.0073	-
	Settling time (s)	1.5392	1.5400	-	1.5260	1.5265	-
	Overshoot (%)	6.4577	12.4754	-	13.4140	8.3308	-
	RMSE (μm)	0.8808	0.8784	-	0.9933	0.9521	-
Ramp 20 μm/s	MAE (μm)	0.1823	0.1826	-	0.2360	0.1340	-
	RMSE (μm)	0.0673	0.0669	-	0.0959	0.0946	-
Ramp 50 μm/s	MAE (μm)	0.4565	0.4558	-	0.3470	0.3360	-
	RMSE (μm)	0.1583	0.1573	-	0.1524	0.1511	-
Sinusoidal 2 Hz	Phase lag (º)	6.480	5.7600	0.0000	4.3200	3.6000	0.0000
	MAE (μm)	0.6648	0.6628	0.0191	0.7283	0.6807	0.3157
	RMSE (μm)	0.4706	0.4690	0.0116	0.4644	0.4581	0.0385
Sinusoidal 5 Hz	Phase lag (º)	16.200	14.400	0.0000	10.800	9.0000	0.0000
	MAE (μm)	1.7288	1.7225	0.1114	1.7879	1.7752	0.5065
	RMSE (μm)	1.2569	1.2513	0.0796	1.1644	1.1590	0.1633

M.E.: model estimation; C.S.: control switching; $C_{pre}$: pre-compensator.

## Data Availability

The original contributions presented in this study are included in the article. Further inquiries can be directed at the corresponding author.

## Appendix A

The system specifications are available at https://reurl.cc/rK1r7r, accessed on 12 November 2025.
